# Determining the Need for Additional Testing With Quantiferon TB Gold in Patients With Positive Tuberculin Skin Tests and a History of BCG Vaccination

**DOI:** 10.7759/cureus.39272

**Published:** 2023-05-20

**Authors:** Antony J Arumairaj, Hansang Park, Fernando Quesada, Brian Altonen, Shobhana Chaudhari, Joseph Mattana, Imnett Habtes

**Affiliations:** 1 Internal Medicine, New York Medical College, Metropolitan Hospital Center, New York, USA; 2 Biostatistics and Epidemiology, New York City Health and Hospitals Corporation, New York, USA; 3 Internal Medicine, Division of Pulmonary and Critical Care, New York Medical College, Metropolitan Hospital Center, New York, USA

**Keywords:** mycobacterium tuberculosis, cross-reactivity, high tb burden, bcg vaccination, latent tuberculosis infection, tuberculin skin test, quantiferon tb gold

## Abstract

Objective: To determine if the QuantiFERON-TB Gold (QFT) testing can be obviated for the diagnosis of latent tuberculosis infection (LTBI) in patients with a positive tuberculin skin test (TST) and a history of Bacillus Calmette-Guerin (BCG) vaccination by identifying high-risk features in patients with positive TST and a history of BCG vaccination who are associated with positive QFT.

Methods: Retrospective chart review was done for 76 adult patients by dividing them into two groups. Group 1 consisted of true positive TST patients who had BCG vaccination and were positive for QFT. Group 2 consisted of false positive TST patients who had BCG vaccination but were negative for QFT. The two groups were compared to determine if the high-risk features of TST induration diameter of 15mm and more, TST induration of 20mm and more, recent immigration to the US, the advanced age of more than 65 years, country of origin with high TB burden, known exposure to active TB, and smoking history were more prevalent in Group 1 compared to Group 2.

Results: Group 1 had 23 patients and Group 2 had 53 patients. Group 1 had a higher prevalence of patients with PPD induration of more than 10mm than Group 2, which was statistically significant with a P value of 0.03. Other risk factors of advanced age, exposure to active TB and smoking did not show statistically significant differences between Groups 1 and 2.

Conclusion: This study also confirms that if the TST induration is more than 10mm in patients with a history of BCG vaccination, the TST induration is likely because of LTBI and is less likely because of cross-reaction with BCG vaccination.

## Introduction

Tuberculosis (TB) is a contagious infection accounting for 1.4 million deaths yearly, more than other infectious diseases, including human immunodeficiency virus (HIV)/ acquired immunodeficiency syndrome (AIDS) [1]. Infection with Mycobacterium tuberculosis (M. tuberculosis) resulting in latent TB infection is the precursor to TB disease [2]. About one-quarter of the world’s population is infected with M. tuberculosis, the pathogen that causes TB [2]. Individuals with latent TB infection (LTBI) show no symptoms. Once infected with LTBI, most people remain asymptomatic and are not contagious. They may carry the infection for months or even years and are at risk for developing active and contagious TB disease [3]. However, 5-10% of those infected may progress to active TB in their lifetime and become infectious [4]. Identifying patients with latent tuberculosis infection (LTBI) and promoting the prophylactic treatment of people with LTBI is critical in controlling the spread of active tuberculosis (TB) infection. Latent TB infections comprise a significant reservoir of future epidemics [3]. 

Administration of the Bacillus Calmette-Guerin (BCG) vaccine is common in children born in countries with a high prevalence of TB to prevent childhood TB meningitis and miliary TB disease [5]. However, BCG is not generally recommended in the United States because of the low risk of infection with M. tuberculosis, the variable effectiveness of the vaccine against adult pulmonary TB, and the vaccine’s potential interference with tuberculin skin test reactivity [6].

People previously vaccinated with BCG may receive a tuberculin skin test (TST) to test for TB infection. One problem in the clinical applicability of the TST is its cross-reactivity with antigens present in BCG vaccine strains and environmental mycobacterial species. This cross-reactivity leads to false-positive results and decreases the positive predictive value of the TST [7]. A positive reaction to a TB skin test may be due to the BCG vaccine or infection with M. tuberculosis [8].

QuantiFERON-TB Gold (QFT) is an interferon-gamma release assay (IGRA) which, unlike the TB skin test, is not affected by prior BCG vaccination and is not expected to give a false-positive result in people who have received the BCG vaccine. QFT detects TB infection by measuring the release of interferon-gamma (IFN-g) from patient T cells after stimulation of a whole blood sample with highly specific M. tuberculosis antigens [9]. Because the test is specific to proteins found in M. tuberculosis, there is no cross-reactivity with the BCG vaccine. QFT is the preferred method of TB testing for people who have received the BCG vaccine [10]. However, implementation of this test has its issues as this is a more expensive test and also because of the need for special equipment and skilled personnel. The cost of QFT is three times higher than the cost of TST. The scarce availability of QFT in resource-limited and high Tb burden nations may affect the ability to detect patients with LTBI [11].

We often have patients referred to our clinic with a positive TST and a history of BCG vaccination. Before starting treatment, we typically refer Quantiferon TB Gold, as the current recommendation by the Centers for Disease Control and Prevention (CDC) is to obtain an IGRA in patients with a positive skin test and previous history of BCG vaccination [12]. However, it is still being determined if there are any high-risk patient characteristics that would obviate the need for additional QFT testing. Based on our analysis, we identified the following high-risk features. Patients with a TST induration diameter of 15 millimeters (mm) and more, and a TST induration diameter of 20mm and more, indicating a positive TST, were considered high-risk factors [13]. Patients who had recent exposure history to tuberculosis in their home country, i.e., immigrated to the United States (US) recently in the past five years, were considered high-risk [14]. Patients who were older than 65 years were considered to be high-risk because of their advanced age [15]. Patients who migrated from high TB burden nations were considered high-risk [16]. Patients with known exposure to active TB were considered high-risk [17]. Patients with a smoking history were considered high risk [18]. The purpose of this study is to analyze if the above-mentioned high-risk patient characteristics as well as a previous history of BCG vaccination have statistical associations with positive QFT to obviate the need for additional QFT testing in this high-risk patient population. We hypothesize that, in patients with these high-risk features, a positive TST is more likely to be indicative of true exposure and less likely to represent cross-reactivity from positive BCG.

This original article was presented as an abstract at the CHEST conference on October 18, 2020.

## Materials and methods

A retrospective chart review was conducted at Metropolitan Hospital in New York, NY. The study included 76 adult patients referred to our pulmonary TB clinic at Metropolitan Hospital with a positive TST history and a history of BCG vaccination between January 2014-January 2020. The study was approved by Brany Institutional Review Board and by the New York City Health & Hospitals Corporation (HHC) central office.

Inclusion and exclusion criteria

Inclusion criteria for the study were adult patients who were 18 years of age and above, patients with positive TST as defined by the CDC, a history of BCG vaccination based on clinical exam or patient reporting, and who underwent a QFT. The exclusion criteria of the study were patients who were less than 18 years of age, patients who had previously completed treatment for LTBI or active TB infection, and patients who were clinically suspicious of active TB infection.

Study design

Patient data was extracted from medical records. The patients were divided into two groups. Group 1 consisted of true-positive TST patients who had BCG vaccination and were positive for QFT. Group 1 consisted exclusively of patients who had LTBI. Group 2 consisted of false-positive TST patients who had BCG vaccination but were negative for QFT. Group 2 consisted of patients who did not have LTBI but were positive for TST because of BCG vaccination. The two groups were compared with each other to determine if the high-risk clinical features were more prevalent in Group 1 compared to Group 2. The high-risk groups of TST in mm, age of patients, recent immigration, exposure to active TB, smoking history and country of origin with high endemicity are further expanded on in the Results section.

Data analysis

This study utilized standard descriptive analysis to analyze and report on continuous variables such as age, years since immigration, and TST mm, focusing on means ± SD. The remaining analyses of categorical variables were carried out using chi-squared and Fisher’s exact tests. Analysis of variances (ANOVA) was employed for gender, race-ethnicity, and country comparisons. Changes that resulted in a p-value of <.05 were considered statistically significant (CI = 95%). All analyses were performed using IBM SPSS Statistics v. 28.0.1.1 (IBM Corp., Armonk, NY). 

## Results

As shown in Table 1, 76 patients qualified for the study after considering the inclusion and exclusion criteria. Of the 76 patients, 61 were female, and 15 were male. There was a strong female preponderance in this study. The vast majority of patients were in the age group of 18-40 years, accounting for 61.8%, followed by those in the age group of 41-65 years, accounting for 34.2%, followed by the age group of more than 65 years, which accounted for 3.9%. The average age of the patients enrolled was 36 years.

**Table 1:** Demographics

Total	Number	%
Age in years, median (IQR)	36.5 (19.75)	
Age in years		
18-40 years	47	61.80%
41-65 years	26	34.20%
66 and > 66 years	3	3.90%
Gender		
Male	15	19.70%
Female	61	80.30%

As shown in Table 2, of the 76 patients who met the criteria for our study, Group 1, which constituted patients with LTBI, had 23 patients and Group 2, which constituted patients who did not have LTBI but were positive for TST because of previous BCG vaccination, had 53 patients.

**Table 2:** Results

High-risk features	Group 1 (23)	Group 2 (53)	P-value
Age In categories			
A (18-40 years)	14 (65.2%)	34 (64.2%)	0.96
B (41-65 years)	8 (34.8%)	17 (34.0%)	1
C (> 65 years)	1 (4.3%)	2 (3.8%)	0.92
Sex			
Female	18 (79.2%)	42 (79.6%)	
Male	5 (20.8%)	11 (20.4%)	
TST induration cutoff categories			
5mm	23 (100%)	52 (98.1%)	0.5
10mm	23 (100.0%)	44 (83.0%)	0.03
15mm	15 (39.1%)	27 (50.9%)	0.25
20mm	14 (60.9%)	20 (37.7%)	0.06
Exposure to active Tb: Yes; No; Not available	2 (8.7%); 19 (82.6%); 2 (8.7%)	3 (5.6%); 39 (73.6%); 11 (20.8%)	0.74
Smoking history: Yes; No; Not available	1 (4.3%); 22 (95.7%); 0 (0.0%)	6 (11.3%); 43 (81.1%); 4 (7.6%)	0.29
Origin			
Mexico	13 (54.2%)	15 (27.8%)	0.02
Caribbean	2 (8.3%)	6 (20.4%)	0.73
Central America	0 (0.0%)	5 (5.4%)	0.12
South America	1 (4.2%)	4 (7.5%)	0.6
Asia	2 (8.3%)	4 (7.5%)	0.86
Africa	3 (12.5%)	6 (11.1%)	0.83
Eastern Europe	0 (0.0%)	1 (1.9%)	0.5
Australia	0 (0.0%)	1 (1.9%)	0.5
United States	1 (4.2%)	2 (3.7%)	0.9
Not available	1 (4.2%)	9 (16.7%)	

The results showed no statistically significant difference between Group 1 and Group 2 in the age groups of 18-40 years and 41-65 years and more than 65 years of age. There was also no statistically significant difference between Group 1 and Group 2 among patients with exposure to active TB and smoking history. There was no statistically significant difference between Group 1 and Group 2 across all categories of recent immigration.

In the evaluation of patients based on TST induration, there was no statistical significance for the TST induration cutoff of positivity at 5mm, at 15mm and at 20mm. For the cutoff of positivity of 20mm, the p-value was 0.06, which trended towards significance. However, for the cutoff of positivity at 10mm, the p-value was significant, with a value of 0.03. This outcome was a significant finding.

In the category of patients based on immigration from regions of TB endemicity, an overwhelming number of patients were from nations with high TB burdens. Mexico constituted the single nation with the highest number of patients in Group 1 (54.2%) and Group 2 (27.8%). The remaining patients were distributed sparsely across different nations of Asia, Africa, and South America, as listed in Table 3. This sparse distribution of patients among the high-TB burden nations showed no statistically significant difference except for patients from Mexico. Patients from Mexico were more prevalent in Group 1 than Group 2, with a statistically significant p-value of 0.02. Patients from USA and Australia were the only nations with low TB burdens in this study. Three patients were born in the USA and met the study criteria, out of which only one had LTBI.

**Table 3:** Nation-wise distribution of patients

Distribution of patients across nations	Group 1	Group 2
Mexico	13	15
Dominican Republic	2	5
Ecuador	1	3
USA	1	2
Guatemala	0	2
Sierra Leone	0	2
India	2	0
Senegal	1	1
Mali	1	1
Australia	0	1
Bangladesh	0	1
Belize	0	1
Brazil	0	1
Egypt	0	1
El Salvador	0	1
Guinea	0	1
Haiti	0	1
Honduras	0	1
Philippines	0	1
Romania	0	1
Togo	0	1
Yemen	0	1
Zambia	1	0
None/NA	1	9
Total	23	53

## Discussion

Addressing the latent TB infection reservoir is critical to achieving TB elimination. As the global community looks to meet ambitious targets for reduction (90% reduction in TB incidence by 2035) and even elimination of TB (less than one incident case per 1,000,000 per year) by 2050, our ability to address the LTBI reservoir will be critical in our chance to succeed [2]. Identifying patients with LTBI in resource-limited, high-TB-burden countries and initiating prophylactic treatment is crucial in achieving TB elimination [19]. The aim of this study was to determine if the high-risk clinical parameters in patients with positive TST and previous history of BCG vaccination were predictive of LTBI to obviate the need for additional QFT testing in this high-risk patient population. The outcome of the study showed that in the evaluation of the high-risk factors, only the TST induration of more than 10mm and immigration from certain regions with a high TB burden had a statistically significant higher prevalence in Group 1 over Group 2.

TST induration of 10mm and more had a significant statistical difference with a p-value of 0.03. But the TST positivity induration cutoff at 15mm and 20mm did not show a definite statistical difference. This study indicates that although the TST induration cutoff of 15mm and 20mm indicated a strongly positive TST, the TST induration of 10mm and more was sufficient to indicate that the positive TST was from true LTBI and not from previous BCG vaccination. This finding indicates that when a patient has a TST induration of 10mm and more, irrespective of whether the patient had the BCG vaccine, it must be considered evidence of LTBI. This finding was consistent with the US Preventive Services Task Force (USPSTF) observation that TST induration of 10 mm and more should not be attributed to prior BCG vaccine [20].

Although several studies have concluded that TST induration cutoffs of 10mm, 15 mm, and 20mm are more likely to be the result of tuberculous infection than of BCG vaccination, a review of national and international childhood TB guidelines found that most countries and agencies use a TST induration cutoff of 10 mm irrespective of BCG vaccination status unless there are immunosuppressive conditions present, in which case the TST induration cutoff of 5mm is acceptable, which is in line with the advice of World Health Organization (WHO) [21].

There is a high prevalence of LTBI in countries with a high burden and endemicity of TB [2]. In our study, there was a large patient group from Mexico, one of the countries with high TB endemicity and burden. There was a definite statistical significance in Group 1 over Group 2, with a p-value of 0.02. This finding indicates that immigration from a country with a high TB burden is a high-risk factor and indicates a higher prevalence of LTBI [22]. Though there were patients from other countries with high TB endemicity, the distribution of the patients across these nations was sparse, as demonstrated in Table 3. This sparse distribution of patients precluded the determination of the association between the origin of countries with high TB endemicity and the prevalence of LTBI.

Advanced age of more than 65 years was considered high risk for LTBI [15]. Older adults remain the largest TB reservoir in the USA, as almost one-quarter of all TB cases are found among those 65 years of age and older [23]. Elderly patients were predisposed to LTBI because of age-associated immune senescence, the use of immuno-modulating drugs, the increasing prevalence of comorbid illnesses of diabetes mellitus and chronic kidney disease, and protein malnutrition [15]. Our study did not show an increased prevalence of LTBI in the elderly patient group of more than 65 years of age.

The effect of the BCG vaccine in causing TST induration is time limited. The effect of BCG vaccination on TST induration wanes with advancing age [24]. It was expected that in patients aged between 18-45 years, there would be a higher prevalence in Group 2, consisting of those with false-positive TSTs, than Group 1, consisting of the LTBI group since the effect of BCG vaccination on TST would have lasted longer in them than in those patients who were older than 65 years (because the impact of infant BCG vaccination on TST responses wanes with age). But the results of the study showed that the patients in the age group 18-45 years were nearly equally distributed in both groups.

Smoking has been a strong risk association with LTBI [18]. Some studies have identified smoking as an independent risk factor for LTBI because of its effect on the lungs and the body’s immune system, causing mechanical disruption of ciliary function and giving rise to multiple defects in the immune cells such as macrophages, monocytes and CD4 lymphocytes, leading to altered immune response [25]. However, the cohort of our patients had a strong Hispanic female preponderance with a low prevalence of smoking in the study population. Smoking did not have a significant statistical significance in Group 1 over Group 2.

Latent TB infection can occur upon contact with active TB patients. The risk is potentially increased with closer exposure intensity. Household members have the most significant risk for latent TB infection because they share the same air with active TB patients for a longer time [26]. However, exposure to active tuberculosis did not have a significant statistical significance in Group 1 over Group 2.

The incidence of tuberculosis among migrants is greatest within the first five years of arrival, driven by health-related factors of age and comorbid status and socioeconomic factors [27]. CDC recommended targeted testing and treatment of LTBI among non-US-born individuals who have been in the United States for five years or less [28]. However, for individuals from high TB-burden regions, the risk for an infection after more than 20 years post-US entry approaches the risk of newly arrived individuals from lower TB-burden regions. USPSTF issued a recommendation in 2023 that individuals from countries with increased TB risk undergo LTBI screening without addressing the length of time since US entry [29]. Our study showed no statistically significant prevalence among immigrants across all age groups in Group 1 over Group 2.

This study confirms that if the TST induration is 10mm and more, irrespective of the status of the BCG vaccination, the TST induration is likely because of LTBI and is less likely because of cross-reaction with BCG vaccination [30]. Although the TST induration cutoff of 15mm and 20mm indicates strongly positive TST, it does not always indicate a high-risk exposure [8]. These findings will be crucial in resource-limited settings worldwide where the burden of LTBI is high and where the availability of QFT might be limited.

There were several limitations in this study. First, the sample size was small, because of which the distribution of patients across various categories and subgroups was limited. Second, there were some missing data in this study present in certain categories. Third, although most patients were vaccinated soon after birth, we could not document exactly when each patient received the BCG. This makes it more difficult to determine how quickly the effects of BCG on TST responses wane. Fourth, we used evidence of a BCG scar, documentation of vaccination, or a clear history of vaccination from the patient as evidence of BCG vaccination. It is possible that some patients were incorrectly classified using this approach. Fifth, this study was conducted in a New York City Hospital, and caution should be exercised when generalizing results to other clinical settings. 

## Conclusions

In the resource-rich setting of a developed world, the findings of TST, especially when the TST induration is more than 10mm and when QFT is available, the QFT may be used in place of, or in addition to, the TST for patients who are known to have received a BCG vaccine for definitive management. However, in resource-limited, high-LTBI burden settings worldwide where the availability of QFT might be limited, the high-risk clinical parameter of TST induration diameter greater than 10mm is predictive of LTBI irrespective of the status of BCG vaccination, thereby obviating the need for additional QFT testing. This finding will be critical in identifying and management of the reservoir of LTBI patients in resource-limited countries with a high TB burden.
